# Molecular radiotherapy for adult type metastatic neuroendocrine tumours in children

**DOI:** 10.1007/s00259-025-07247-6

**Published:** 2025-04-24

**Authors:** Connie Peet, Caroline Elmaraghi, Tarek Abdel-Aziz, Huang Hian Liang, Jennifer E. Gains, Trung Nguyen, Simon Wan, Jamshed B. Bomanji, Mark N. Gaze

**Affiliations:** 1 Department of Radiotherapy, University College London Hospitals NHS Foundation Trust, London, UK; 2 Department of Oncology, University College London Hospitals NHS Foundation Trust, London, UK; 3 Department of Endocrine Surgery, University College London Hospitals NHS Foundation Trust, London, UK; 4 Department of Nuclear Medicine, University College London Hospitals NHS Foundation Trust, London, UK; 5 Department of Paediatric Oncology, University College London Hospitals NHS Foundation Trust, London, UK

**Keywords:** Lutetium DOTATATE, Meta iodobenzylguanidine, Molecular radiotherapy, Neuroendocrine tumours, Paediatric, Phaeochromocytoma and paraganglioma

## Abstract

**Purpose:**

Paraganglioma, phaeochromocytoma and gastroenteropancreatic neuroendocrine tumours are rare in childhood. Molecular radiotherapy is one potential treatment for locally inoperable or metastatic disease. This study reviews the use and efficacy of molecular radiotherapy with both [^131^I] meta iodobenzylguanidine (mIBG) and [^177^Lu] DOTATATE in this patient group.

**Methods:**

This is an observational cohort study of all patients aged less than 18 years with adult type metastatic neuroendocrine cancers treated with molecular radiotherapy from 2003 to 2023 in one national referral centre.

**Results:**

Twelve patients, six male and six female, were treated. The median age at diagnosis was 12 years 3 months (range 7 years 11 months to 15 years 5 months), and at first molecular radiotherapy treatment was 13 years 7 months (range 8 years 8 months to 16 years 2 months). Nine had paraganglioma or phaeochromocytoma, three had other neuroendocrine tumours. Three received [^177^Lu] DOTATATE only, four received [^131^I] mIBG only, and five received both radiopharmaceuticals. Three patients had rapid disease progression and died within a year. Following initial treatment of the others, two had a complete response, four had a partial response, one had stable disease, and two had a mixed response. Nine patients remain alive, at a median of 5 years 0 months (range 2 years 4 months to 21 years 5 months) after start of treatment.

**Conclusion:**

Molecular radiotherapy can be beneficial, and may provide good disease control for long periods in a proportion of these patients. Combining different radiopharmaceuticals may be of value.

## Introduction

Paraganglioma and phaeochromocytoma (PPGL), and other malignant neuroendocrine tumours (NET) occur predominantly in adult life, but may also rarely present in childhood. The incidence is so low in paediatric practice, that they do not have distinct and separate recognition in a major population-based epidemiological report of malignant disease in children, teenagers and young adults, but are grouped as “other and unspecified malignant neoplasms” [1]. The annual incidence of PPGL is reported to be between 0.5 and 2.0 cases per million children [2]. The incidence of NET in children and young people (age 0 to 29 years) is estimated as 2.8 (95% confidence intervals 2.6 to 3.0) per million person years [3].

For patients with operable, localised disease, surgery is a potentially curative treatment [4]. Metastatic disease predicts a poorer prognosis [5]. Around one third of patients with PPGL carry an underlying susceptibility gene which may influence disease aggression and metastatic potential. Patients with a tumour suppressor mutation like *VHL* or a *RET* proto-oncogene are less likely to develop malignant disease although further disease may occur in the contralateral adrenal gland. A less aggressive surgical approach like cortical-sparing adrenalectomy maybe possible in these patients to preserve cortisol secretion and reduce risk of life-threatening Addisonion crisis if a bilateral adrenalectomy is necessary. This is especially important as the teenage and young adult population may have relatively poorer compliance to medication [6–8]. 

On the other hand, children with PPGL arising on the basis of a germline succinate dehydrogenase subunit B (*SDHB*) mutation, are more likely to have metastatic disease and may require radical surgery with some form of adjuvant systemic therapy [9].

PPGL is therefore an example of where precision medicine is applicable: a genetic driver mutation may well be identified guiding prognosis; biochemically functionality may result in blood or urine tumour markers for diagnosis, response assessment and surveillance; various nuclear medicine imaging modalities may identify potential therapeutic targets. Collectively, this information and these tools may allow clinicians to tailor an individualised treatment strategy, timing, extent, and to manage patient expectations, especially in cases where disease is deemed non-curable and with tendency to run a long-term remitting, relapsing course.

While observation without immediate treatment may be reasonable for asymptomatic or minimally symptomatic patients with low volume metastatic disease, systemic treatment will be indicated for disease progression. While some chemotherapy options are available, most experience has been in adult patients and limited to retrospective case series and few single-arm prospective trials [10]. Moreover, in a previous cohort, no patients who tested positive for SDHB mutations responded to chemotherapy [11], suggesting the need for alternative therapies particularly in the paediatric population. Molecular radiotherapy is recognised as a valuable treatment for those patients with metastatic disease where nuclear medicine imaging demonstrates good uptake of the diagnostic radiotracer for a particular therapeutic radiopharmaceutical [2].

[^131^I] meta iodobenzylguanidine (mIBG) is taken up by tumour cells of neural crest origin which express the human noradrenaline (norepinephrine) transporter [12]. This radiopharmaceutical was first used to treat phaeochromocytoma more than 40 years ago [13]. Since then, the encouraging outcomes of large series of adult patients with metastatic phaeochromocytoma treated with [^131^I] mIBG have been reported [14].

More recently, peptide receptor radionuclide therapy has been developed for the treatment of metastatic neuroendocrine tumours which express somatostatin receptors [15]. Various radionuclides including ^90^Y, ^177^Lu and ^225^Ac, conjugated with various octreotide analogues including DOTATOC and DOTATATE have been used [16, 17]. This form of molecular radiotherapy has been shown to be effective in the treatment of adult patients with both metastatic gastroenteropancreatic NET and PPGL [18, 19].

However, despite the many reports of molecular radiotherapy for metastatic PPGL, gastroenteropancreatic and other NET in adults, there is very little in the medical literature about the use of these treatments specifically in children. Reported series have small numbers, no doubt due to the rarity of these tumour types in the paediatric population.

Reports of the use of mIBG therapy in children with tumours other than neuroblastoma are very limited. There are case reports and series with one, two, three, seven and eight patients [20–24].

The use of yttrium DOTATOC ([^90^Y] DOTATOC) in six patients aged under 18 years has been reported [25]. The safety and efficacy of lutetium DOTATATE ([^177^Lu] DOTATATE) therapy with neuroendocrine cancers have been reported in series of two and eight children [26, 27]. A prospective clinical trial (ClinicalTrials.gov ID NCT04711135) to evaluate the safety and dosimetry of [^177^Lu] DOTATATE in adolescent patients with gastroenteropancreatic NET and PPGL has completed recruitment of 11 patients.

This paper reports a single institution retrospective cohort study of 12 patients with metastatic PPGL or other adult type NET (except neuroblastoma) aged less than 16 years at the time of first molecular radiotherapy treatment with either [^131^I] mIBG or [^177^Lu] DOTATATE. The aim is to describe treatment, response and survival, and propose new concepts for future practice.

## Patients and methods

All patients aged less than 18 years at the time of first treatment with molecular radiotherapy for metastatic adult-type neuroendocrine tumours between 2003 and 2023 at University College London Hospitals (UCLH) National Health Service (NHS) Foundation Trust (FT) were included. This cohort did not include children with neuroblastoma, or three subsequently treated patients who were enrolled in a clinical trial (NCT04711135) which will be reported separately. UCLH NHS FT sponsored this research, which was approved by a Research Ethics Committee, and conducted according to a written protocol. Patients were identified from departmental records, and data were extracted from clinical records.

At the start of the study period, only [^123^I] mIBG scanning was available for disease assessment, but over the most recent 15 years, [^68^Ga] gallium DOTATATE positron emission tomography (PET) computed tomography (CT) has been part of our standard of care evaluation of children and young people with metastatic neuroendocrine tumours. Typically, we perform triple imaging assessment at baseline, with [^18^F] fluorodeoxyglucose (FDG) PET CT, [^123^I] mIBG planar scintigraphy with single photon emission computed tomography (SPECT) CT and [^68^Ga] gallium DOTATATE PET CT. Patients are discussed in a molecular radiotherapy muti-disciplinary team meeting with nuclear medicine physicians, surgeons and paediatric and clinical oncologists, and individualised decision making is performed on the basis of clinical parameters, imaging results and radiopharmaceutical availability. Treatment starts as soon as practicable after baseline assessments, usually within six to eight weeks.

[^131^I] mIBG was administered intravenously through a central venous catheter over approximately 30 min following four hours prehydration and administration of prophylactic antiemetics. Hydration was continued for a total of 24 h. [^177^Lu] lutetium DOTATATE was administered intravenously through a central venous catheter over approximately 30 min, concomitantly with an amino acid solution to reduce renal radiation exposure [28].

Given the heterogeneous nature of the patient population and treatments given, with no comparators, the analysis was purely descriptive of the observations.

## Results

### Patient characteristics

Twelve patients, six male and six female were treated. The median age at diagnosis was 12 years 3 months (range 7 years 11 months to 15 years 5 months). The median period between diagnosis and start of molecular radiotherapy was 9 months (range 2 months to 3 years three months). The median age at first treatment was 13 years 7 months (range 8 years 8 months to 16 years 2 months). Table 1 shows selected patient characteristics, treatments given, and outcomes.

The histopathological diagnosis was phaeochromocytoma in two patients, paraganglioma in seven patients, gastroenteropancreatic NET in two patients (one pancreatic insulinoma, and one carcinoid tumour with unknown primary site), and malignant tumour with neuroendocrine differentiation (thymic primary tumour associated with ectopic adrenocorticotrophic hormone production and Cushing syndrome) in one patient.

The tumour arose on the background of a genetic predisposition syndrome in many patients. SDHB mutations were documented in five of the nine phaeochromocytoma and paraganglioma patients, and patient with an insulinoma had multiple endocrine neoplasia type 1 (MEN1). The cause was unknown in the other patients.

**Table 1:** Details of the twelve patients

Diagnosis	Genetics	Age at diagnosis	Treatment prior to MRT	Age at first MRT	MRT used	Response	TTP	Further treatment	TTP	Further treatment		Outcome
PGG	SDHB	7y 11 m	Surgery RT	11y 2 m	mIBG x 6	PR	-	-	-	-		Alive 2y 4 m
PGG	SDHB	15y 1 m	Surgery	15y 3 m	LuDO x 4	SD	1y 1 m	mIBG x 6	7y 10 m	RT Chemo		Alive 11y 9 m
PGG	-	11y 4 m	Surgery	14y 1 m	LuDO x 4	MR	3y 10 m	mIBG x 3 (ongoing)	-	-		Alive 4y 4 m
PGG	SDHB	12y 8 m	-	12y 10 m	mIBG x 6	PR	4y 5 m	RT mIBG x 6	-	-		Alive 8y 5 m
PGG	-	7y 11 m	Chemo Surgery	8y 8 m	mIBG x 1	CR	6y 0 m	LuDO x 4 Surgery RT	-	-		Alive 21y 5 m
PGG	SDHB	11y 11 m	Octreotide analogues	12y 4 m	mIBG x 6	MR	2y 0 m	RT LuDO x 4	4y 7 m	-		Alive 7y 6 m
PGG	-	8y 5 m	Surgery	9y 2 m	mIBG x 4	PD	6 m	LuDO x 2	4 m	-		Died 1y 0 m
PCC	-	13y 0 m	Surgery	14y 2 m	mIBG x 4	PD	6 m	-	-	-		Died 11 m
PCC	SDHB	14y 10 m	Surgery	15y 2 m	mIBG x 6	PR	-	-	-	-		Alive 10y 7 m
NET Insulinoma	MEN1	10y 7 m	Surgery Octreotide analogues	13y 2 m	LuDO x 4	CR	1y 7 m	LuDO x 4	-	-		Alive 5y 8 m
NET Carcinoid	-	15y 5 m	Octreotide analogues	16y 2 m	LuDO x 3	PR	-	-	-	-		Alive 2y 10 m
NET Thymus	-	14y 4 m	Octreotide analogues	15y 2 m	LuDO x 3	PD	4 m	-	-	-		Died 9 m

Abbreviations: Chemo– chemotherapy; CR– complete response; LuDO– [^177^Lu] DOTATATE; mIBG– [^131^I] meta iodobenzylguanidine; m– months; MEN1– multiple endocrine neoplasia type 1; MR– mixed response; MRT– molecular radiotherapy; NET– neuroendocrine tumour; PCC– phaeochromocytoma; PD– progressive disease; PGG– paraganglioma; PR– partial response; RT– radiotherapy; SD– stable disease; SDHB– succinic dehydrogenase B mutation; TTP– time to progression; y– years;

### Treatment prior to molecular radiotherapy

Eight patients had surgery as part of initial treatment after diagnosis. In three of these patients, there followed a period of active surveillance until the development of metastatic disease in two who had initially localised and fully resected disease, and progression of low volume metastatic disease present at the time of diagnosis in the other. One patient had neoadjuvant chemotherapy before surgery. One patient received external beam radiotherapy to vertebral disease threatening spinal cord compression, after debulking surgery.

The other four patients were referred for molecular radiotherapy soon after diagnosis and staging investigations. Patients also received non-cytotoxic supportive care medication to improve their symptoms and general condition as necessary including anti-hypertensives, analgesics, octreotide analogues, insulin and metyrapone.

### Molecular radiotherapy treatment, response and retreatment

[^131^I] mIBG was the first molecular radiotherapy treatment for seven of the nine phaeochromocytoma and paraganglioma patients. Of these, one patient received one course of adjuvant [^131^I] mIBG following neoadjuvant chemotherapy and surgery for a node positive bladder paraganglioma. Two patients received four courses of mIBG before treatment was terminated earlier than planned because of progressive disease. The other four patients received six courses at two-month intervals. The prescribed activity per course was 7.4 GBq in three patients, and 11.1 GBq in one patient, based on their size and age.

Two patients with paraganglioma each received four courses of 7.4 GBq [^177^Lu] DOTATATE at two-month intervals as their initial treatment.

The patient who received chemotherapy, surgery and a single administration of mIBG was well with no evidence of disease for 5 years 10 months, when she developed extensive pelvic lymph node recurrence. She was then treated with four courses of [^177^Lu] DOTATATE, radical surgery and external beam radiotherapy, and remains disease free more than 20 years after her first treatment.

Of the two patients whose mIBG treatment was stopped after four administrations because of progressive disease, one received two courses of [^177^Lu] DOTATATE with no benefit, the other received no further treatment. Both died from progressive disease.

**Fig. 1:**
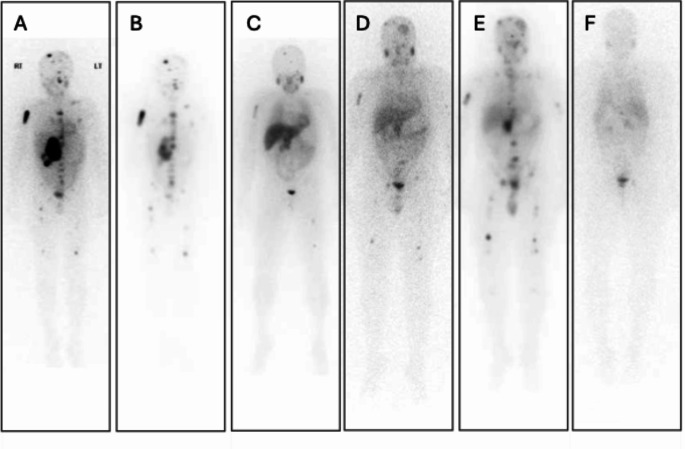
Images of a patient aged 12 years 8 months when diagnosed with a metastatic abdominal paraganglioma, to show extent of disease before and after six initial courses of [^131^I] mIBG therapy, and before and after six further courses for relapse. **A**: December 2015 [^123^I] mIBG diagnostic whole body planar scan at diagnosis; **B**: January 2016 Whole body planar scan following first [^131^I] mIBG therapy; **C**: September 2017 [^123^I] mIBG diagnostic whole body planar scan after six courses of [^131^I] mIBG therapy; **D**: September 2021 [^123^I] mIBG diagnostic whole body planar scan showing disease progression; **E**: December 2021 Whole body planar scan following first [^131^I] mIBG therapy for relapse, after external beam radiotherapy to skull metastasis; **F**: January 2025 [^123^I] mIBG diagnostic whole body planar scan for follow-up 3 years 3 months after the last of six mIBG treatments for relapse

Of the four patients with PPGL who received the planned six administrations of [^131^I] mIBG, two had a partial response followed by disease stability which has continued for 2 years 4 months and 10 years 7 months so far. One had a partial response which lasted for 4 years 5 months before disease progression, which required further treatment with external beam radiotherapy to a prominent metastasis followed by a further six courses of [^131^I] mIBG therapy. Figure 1 illustrates this patient’s course through treatment. The other patient had a mixed response with clear progression after two years treated with external beam radiotherapy followed by four courses of [^177^Lu] DOTATATE. This resulted in a partial response followed by disease stability for 4 years 7 months before progression.

The two paraganglioma patients who received [^177^Lu] DOTATATE as first line therapy had disease progression after 1 year 1 month and 3 years 10 months. Both went on to receive second line mIBG therapy. The first had a partial response then disease stability for 7 years 10 months before disease progression necessitated further treatment with external beam radiotherapy and chemotherapy. The second is continuing [^131^I] mIBG therapy at present.

The initial molecular radiotherapy for the three patients with other metastatic NET was intended to be four courses of 7.4 GBq [^177^Lu] DOTATATE at two-month intervals. However, only one patient received all four courses. One stopped treatment after three courses because of haematological toxicity. The other received only two courses before tumour progression led to treatment being discontinued.

**Fig. 2:**
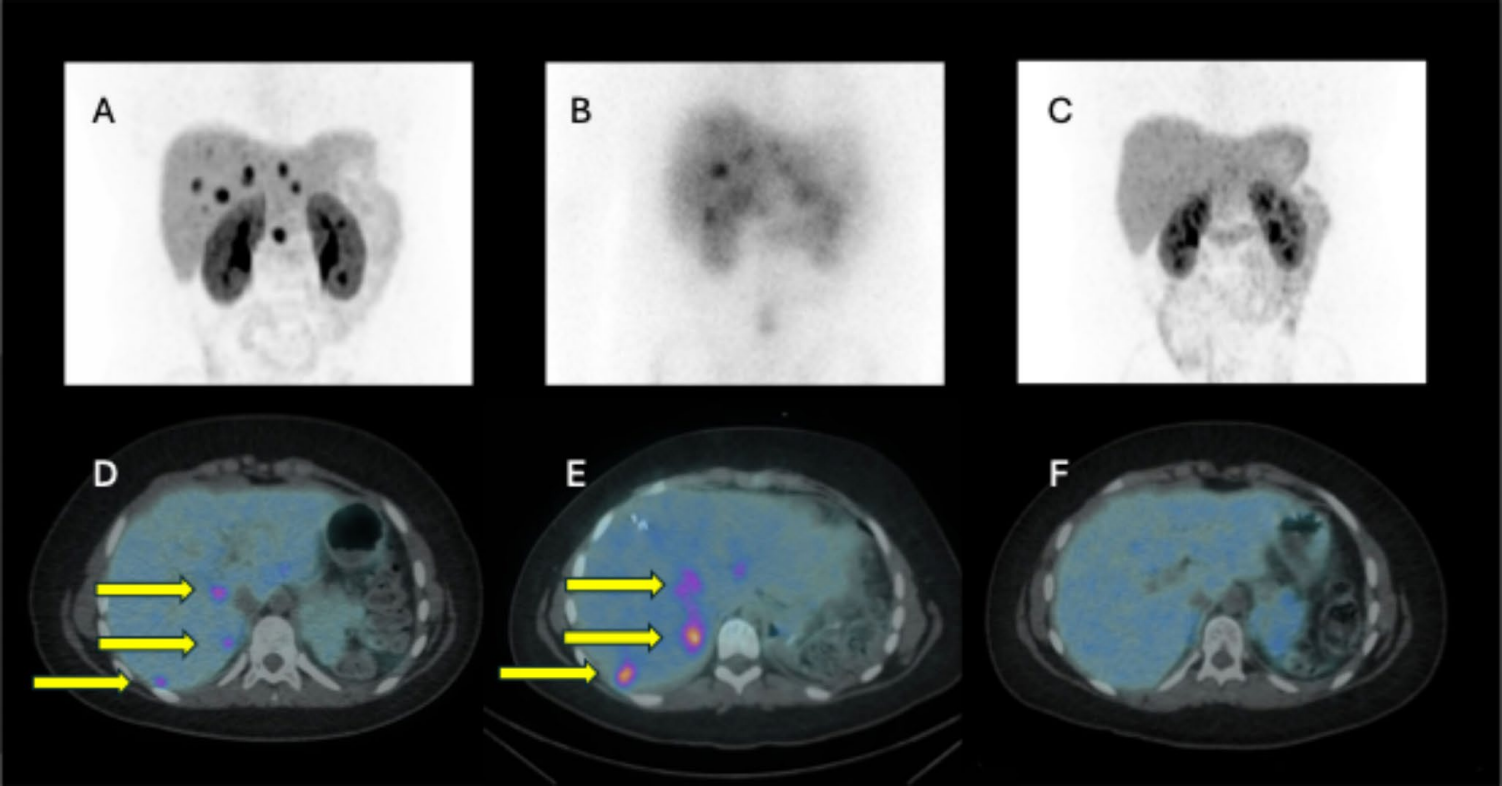
Images of a patient aged 13 years 2 months at treatment for liver metastases from a pancreatic neuroendocrine tumour. **A**: December 2018 upper abdominal maximum intensity projection (MIP) [^68^Ga] DOTATATE PET CT showing metastatic deposits; **B**: February 2019 SPECT MIP following [^177^Lu] DOTATATE therapy demonstrating therapeutic radiopharmaceutical uptake in metastases; **C**: January 2020 follow-up MIP [^68^Ga] DOTATATE PET CT of upper abdomen showing response to treatment; **D**: axial [^68^Ga] DOTATATE PET CT at the same timepoint as panel A through the liver showing metastatic deposits (arrowed); **E**: [^177^Lu] DOTATATE SPECT CT image at the same timepoint as panel B showing uptake of the therapeutic radiopharmaceutical in metastases (arrowed); **F**: follow-up axial [^68^Ga] DOTATATE PET CT at the same timepoint as panel C illustrating imaging response to therapy

The patient with metastatic insulinoma who received four courses of lutetium DOTATATE had a complete response by imaging criteria which lasted for 1 year 7 months. Retreatment with a further four courses of Lutetium DOTATATE led to a second complete remission which has endured for 4 years 1 month so far (Fig. 2).

In summary, seven patients received [^131^I] mIBG, and five were administered [^177^Lu] DOTATATE, as initial therapy. Three of the seven who received [^131^I] mIBG subsequently had [^177^Lu] DOTATATE as second line therapy. Two of the five who received [^177^Lu] DOTATATE later got [^131^I] mIBG as second line therapy. So of the twelve patients, five received both radiopharmaceuticals, four had [^131^I] mIBG only, and three had [^177^Lu] DOTATATE therapy only.

### Survival outcome

Three patients: one thymic neuroendocrine tumour, one phaeochromocytoma and one paraganglioma; failed to respond to treatment and died from rapidly progressive disease after commencing molecular radiotherapy at 9, 11 and 12 months, respectively.

**Fig. 3:**
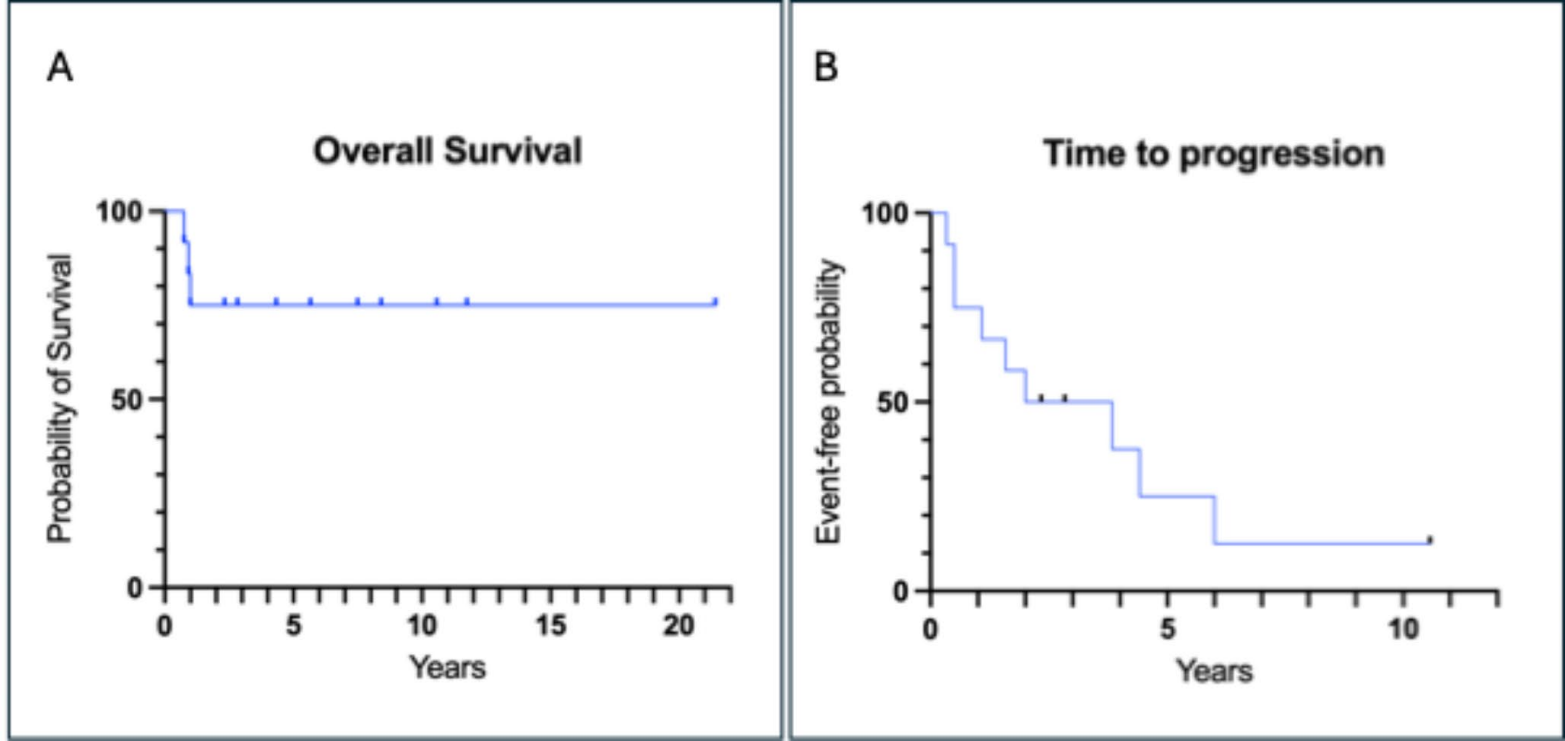
Kaplan-Meier curves of the whole cohort of 12 patients. **A**: overall survival. **B** time to first progression

The other nine patients are alive, at a median of 5 years (range 2 years 4 months to 21 years 5 months) after their first molecular radiotherapy treatment. Figure 3 shows Kaplan-Meier curves demonstrating overall survival and time to first evidence of tumour progression (event-free survival).

### Treatment related morbidity

In general treatment was well tolerated. No renal toxicity was observed. Myelosuppression was generally mild and transient; however, one patient could not receive the fourth planned administration of [^177^Lu] DOTATATE because of prolonged thrombocytopenia. One patient developed myelodysplastic syndrome following eight courses of [^177^Lu] DOTATATE, which required treatment with a bone marrow transplant. There has been no treatment related mortality.

## Discussion

This study has several limitations. Patient numbers were inevitably small. The case-mix was heterogeneous in terms of histology, genetics and clinical syndromes. As a retrospective study over two decades, there was no standardised treatment protocol: treatments were individualised depending on imaging results, and multidisciplinary team opinions. Availability of reimbursement for [^177^Lu] DOTATATE varied across the time period of the study, and this had a significant impact on treatment choice along the course of some of the patients presented. From time to time, irregularities in the supply of [^131^I] mIBG affected scheduled delivery of treatment. Tumour and normal organ dosimetry were not performed.

Despite these limitations, some useful conclusions can be drawn. Firstly, not every patient we treated benefited from molecular radiotherapy as some tumours are relatively resistant to treatment and rapidly progressive. Potentially, routine tumour dosimetry might have identified patients with relatively poor uptake and retention of the radiopharmaceutical, and allowed an ineffective and therefore futile treatment to be discontinued sooner before disease progression. Most patients, however, have good long term survival probabilities, sometimes more than a decade, as a result of molecular radiotherapy and other treatments. Figure 3B shows that most patients’ disease progressed at some time point after initial molecular radiotherapy, with a median event free survival of two years. However, retreatment with molecular radiotherapy after disease progression, sometimes used in conjunction with other treatments, might result in longer periods of disease stability than occurred following the initial treatment. This is demonstrated in Fig. 3A, where the overall survival is 75% at 5, 10 and 20 years (albeit with fewer patients at risk later on). While at the commencement of treatment, there were no plans to use both [^131^I] mIBG and [^177^Lu] DOTATATE in any patient, five patients ended up having both radiopharmaceuticals sequentially.

We have previously shown in neuroblastoma that heterogeneity exists in molecular radiotherapy target expression at both macroscopic and microscopic levels [29, 30]. If the same holds true for adult type metastatic NET in children, then there may be a rationale for electively targeting both the somatostatin receptor and the noradrenaline transporter in patients whose tumours show uptake of both [^68^Ga] DOTATATE and [^123^I] mIBG on pre-treatment diagnostic imaging. This approach has been suggested and explored in early-phase clinical trials [31, 32]. The current approach at our centre is to perform both [^123^I] mIBG and [^68^Ga] DOTATATE scans given the potential differences in the somatostatin receptor and the noradrenaline transporter expressions of these tumours.

While we did not perform dosimetry, its use has been advocated to allow increased personalisation and optimisation of molecular radiotherapy [33–35].

To improve future clinical practice and outcomes, we have undertaken a patient and public involvement and engagement exercise [36]. We recommend international collaboration to establish multicentre clinical trials with adequate patient numbers treated with a standard protocol using both mIBG and lutetium DOTATATE in an individualised manner as guided by clinical features, diagnostic imaging and dosimetric evaluation.

## Data Availability

As this paper reports a single-centre clinical study of very rare diseases with a very small number of patients, data will not be shared because of the reasonable likelihood that individual patients could be re-identified.

## Declarations

### Reporting of observational studies

The STROBE guidance on the reporting of cohort studies has been followed in this paper.
